# Andrew Boydston answers questions about additive manufacturing

**DOI:** 10.1038/s41467-020-17722-3

**Published:** 2020-08-17

## Abstract

Andrew J. Boydston is the Yamamoto Family Professor of Chemistry at the University of Wisconsin-Madison. As a trained chemist he worked on catalysts for the synthesis of polymers during his postdoc time and started his independent career as an assistant professor of Chemistry in 2010 at the University of Washington. In 2014 he involved in a project with colleagues at the mechanical engineering department at the University of Washington which piqued his interest in additive manufacturing and which remained one of his research lines after moving to the University of Wisconsin-Madison in 2018. He is interested in organocatalysts for polymerization reactions, mechanophores, polymers for controlled release and additive manufacturing.

Tell us a little bit about you and what sparked your interest in additive manufacturing.

I think from my personal perspective additive manufacturing seems like an area that benefits from chemistry. As a chemist I am excited to think about this as an opportunity for polymers and functional materials to play a new role. I actually did not have any familiarity with additive manufacturing in any of my academic upbringing. I started my professorship in 2010 at the University of Washington and got involved in additive manufacturing in 2014. Two colleagues at the mechanical engineering department (Professors Duane Storti and Mark Ganter) at the University of Washington approached me with a project and it was clear that the project needed some perspective from chemical synthesis. This piqued my interest purely from the standpoint to address this unmet need. Starting from this we still work together and every time I am talking with them we come up with new ideas. My interest in additive manufacturing has grown naturally based on collaboration in cross-disciplinary discussions. I find additive manufacturing is absolutely a cross-disciplinary field. One of the things, that has been really exciting to me, has been for example going to conferences and seeing presenters from probably the broadest range of different disciplines from any conferences that I was normally going to attend: Anything from simulations work, modelling, mechanical engineering in various aspects, material science development, synthetic chemistry, medicine, dental, it is incredible. I think additive manufacturing is serving as a nice centrepiece for bringing people from different disciplines together to try to establish some common language around big problems.

How has additive manufacturing changed your field? Is there anything specific that it has enabled or made possible? Tell us how it impacted your research.

I think it is slower to impact chemistry to be honest. Some of the things I have seen have been bidirectional in a sense that chemistry has offered to 3D printing but then 3D printing has enhanced chemistry. These are probably things like customization of reactors especially for benchtop scale. I think there is probably also a lot of unrecognized behind the scenes day-to-day assistance that happens just from the fact that when 3D printing hit the latest popularity boom it became also a lot more common to have maker spaces in departments and in individual labs to have 3D printers. I know that several labs with different research focus use standard benchtop 3D printers to produce lab equipment or parts and joints. So, in short, I think there is an underlying assistance that comes from 3D printing, but I think that there has been slow bidirectional enhancement with chemistry.

In our research it has impacted two things: One, I think it has really broadened our thinking in terms of potential applications for the work that we do, and two, it also introduced us to a lot of collaborators in academia and in industry that we probably would not have had access to or had thought to engage without having been looking at 3D printing as an application area. As a result now, our team members are getting a lot broader education as students and as post-docs.

What does additive manufacturing have to do to be more widely adopted? Are there any specific hurdles it has to overcome?

I think that to some extent additive manufacturing needs to establish greater reliability and I think understanding entirely the full process and how to understand part performance completely from the process history is still something that is in need of development. When looking at the field as a chemist I think that the materials development in general is still severely lacking. One becomes compromised as one moves through different types of additive manufacturing and the additive manufacturing methods become application specific for reasons that are centered around limitations in most cases. I think a lot of these limitations come about from inferior materials properties. But I think another issue in this is going to be accelerated by a broader multidisciplinary approach in additive manufacturing. I think the perspective of additive manufacturing in some cases as a design changer is also important. My point of that is that looking at the entire design process and doing that from the perspective of all of the different disciplines that contribute to additive manufacturing is something that has to be adopted.

Looking forward: where do you see additive manufacturing going next?

I am almost hesitant to say. I think while 3D printing is making a lot of progress towards replacing many types of manufacturing for things that already exist, I cannot begin to envision the things that do not yet exist that additive manufacturing is going to make possible. I can start to imagine things that have multi material and multi functionality to them that cannot be created outside of natural biological synthetic pathways or adaptive material systems like personalized medicine, implants and prosthesis that actually age with the patient. I can see additive manufacturing playing a key role in areas like that.

For you, is there a difference between additive manufacturing and 3D printing? If they are not equivalent, how do they differ?

I think that I fall into the trap of using those essentially interchangeably. I think as the community got larger quickly the lines got blurred but I would say that 3D printing is a component of additive manufacturing.

